# Cohort Profile: Resilience, Ethnicity and AdolesCent mental Health (REACH)

**DOI:** 10.1093/ije/dyac051

**Published:** 2022-03-28

**Authors:** Gemma Knowles, Charlotte Gayer-Anderson, Rachel Blakey, Samantha Davis, Katie Lowis, Daniel Stanyon, Aisha Ofori, Alice Turner, Lynsey Dorn, Schools Working Group, Young Persons Advisory Group, Stephanie Beards, Vanessa Pinfold, Ulrich Reininghaus, Seeromanie Harding, Craig Morgan

**Affiliations:** Health Service and Population Research Department, Institute of Psychiatry, Psychology and Neuroscience, King’s College London, London, UK; ESRC Centre for Society and Mental Health, King’s College London, London, UK; Health Service and Population Research Department, Institute of Psychiatry, Psychology and Neuroscience, King’s College London, London, UK; ESRC Centre for Society and Mental Health, King’s College London, London, UK; Health Service and Population Research Department, Institute of Psychiatry, Psychology and Neuroscience, King’s College London, London, UK; ESRC Centre for Society and Mental Health, King’s College London, London, UK; Health Service and Population Research Department, Institute of Psychiatry, Psychology and Neuroscience, King’s College London, London, UK; ESRC Centre for Society and Mental Health, King’s College London, London, UK; Health Service and Population Research Department, Institute of Psychiatry, Psychology and Neuroscience, King’s College London, London, UK; Health Service and Population Research Department, Institute of Psychiatry, Psychology and Neuroscience, King’s College London, London, UK; Health Service and Population Research Department, Institute of Psychiatry, Psychology and Neuroscience, King’s College London, London, UK; Health Service and Population Research Department, Institute of Psychiatry, Psychology and Neuroscience, King’s College London, London, UK; ESRC Centre for Society and Mental Health, King’s College London, London, UK; Health Service and Population Research Department, Institute of Psychiatry, Psychology and Neuroscience, King’s College London, London, UK; ESRC Centre for Society and Mental Health, King’s College London, London, UK; ESRC Centre for Society and Mental Health, King’s College London, London, UK; ESRC Centre for Society and Mental Health, King’s College London, London, UK; Health Service and Population Research Department, Institute of Psychiatry, Psychology and Neuroscience, King’s College London, London, UK; National Children’s Bureau, London, UK; McPin Foundation, London, UK; Department of Public Mental Health, Central Institute of Mental Health, Medical Faculty Mannheim, University of Heidelberg, Mannheim, Germany; Department of Nutritional Sciences, School of Life Course Sciences, Faculty of Life Sciences & Medicine, King’s College London, London, UK; Health Service and Population Research Department, Institute of Psychiatry, Psychology and Neuroscience, King’s College London, London, UK; ESRC Centre for Society and Mental Health, King’s College London, London, UK

Key features

- REACH (Resilience, Ethnicity, and AdolesCent Mental Health) is an accelerated cohort study that was established to examine the extent, nature, and development of mental health problems among young people from diverse social and ethnic backgrounds and densely populated urban areas.
- Three representative cohorts of young people were recruited from mainstream secondary schools in inner-city London [*n* = 4353 of 4945 invited (88%); age 11-14 years, 85% from minority ethnic groups]. Baseline assessments (T1) took place between February 2016 and January 2018.
- The cohorts have been followed up 1 year (T2) and 2 years (T3) later. All 12 schools and over 4000 young people (>90%) remain in the study at T3. An online wave of data collection (T4) is ongoing. Funding has been secured for further follow-ups.
- The dataset comprises a wide range of information on mental health, putative risk and protective factors, and demographics and social circumstances. Linkages to data routinely collected by schools is ongoing. For a nested subsample, further information on mental health, social experiences and circumstances, social cognition, neurocognition, and hypothlamic-pituitary-adrenal (HPA) axis activation (cortisol from hair samples) is available.
- To request access to REACH data for research purposes, and to discuss potential collaborations, please visit [https://www.thereachstudy.com/information-for-researchers.html] or email the lead investigator, Prof. Craig Morgan, at [craig.morgan@kcl.ac.uk].

## Why was the cohort set up?

REACH (Resilience, Ethnicity, and AdolesCent Mental Health) is a school-based accelerated cohort study in inner-city London, UK. REACH comprises three cohorts which were established to provide detailed and extensive information on the nature, distribution, and determinants of mental health among young people from diverse backgrounds and densely populated inner-city areas. Specifically, REACH is designed to test several hypotheses concerning: (i) the extent, nature, and development of mental health problems among young people; (ii) variations by gender, ethnic group, and socioeconomic status; (iii) risk and protective factors; and (iv) mechanisms linking risk/protective factors and mental health. The first phase of REACH, early-to-mid adolescence (2015-20), was funded by the European Research Council. The second phase (2020-21), funded by United Kingdom Research and Innovation (UKRI), examines the impacts of the covid-19 pandemic. The third phase (2021-25), funded by the UK Economic and Social Research Council, will examine the transition to adulthood.

Mental health problems are a major public health issue, with an estimated lifetime prevalence of around 25%.^1^ In the UK, depressive and anxiety disorders rank among the six leading causes of disability among men and women, and substance use disorders rank among the 10 leading causes among men.^2^ In 2009/10, the estimated total economic and social costs of mental ill health in England was £105.2 billion.^3^ There is an urgent need to better understand the development of mental health problems and how to prevent them.

Adolescence is a critical period in the development of mental health problems. Around 50% of mental health problems begin by age 14 and 75% by age 24.^4^ Those who develop recurring or persistent problems during adolescence are at increased risk of a range of adverse social, economic, and health outcomes later in life.^5–7^ In England, the most recent national survey suggests a prevalence of around 14% among those aged 11-16 years.^8^ Data from national surveys and from other major cohort studies in the UK suggest that, for some groups of young people and some types of problems—most notably, depression and anxiety among young women aged 16-24 years—prevalence has increased over the past 15-20 years.^8^^,^^9^ However, for some groups and other types of problems, the data suggest little change over time^8^—which is somewhat surprising against a backdrop of rapid and far-reaching social change (e.g. recession and austerity, rapid increases in the use of mobile technologies, social media, etc.).

Invariably, the most disadvantaged and at-risk groups in society—i.e. most minority ethnic groups, those who grow up in poverty and in challenging circumstances, etc.—are under-represented in national and other large-scale studies.^8^^,^^10^^,^^11^ Indeed, few prospective studies in the UK are sufficiently powered to make meaningful inferences about mental health trends and trajectories among those from minority ethnic groups. This is important because mental health is intimately connected to social, economic, and environmental contexts and experiences. Rates vary by geographical location, socioeconomic status, and ethnic group,^2^^,^^12^ and risk is strongly associated with adverse experiences—discrimination, maltreatment, exposure to crime and violence, etc—which disproportionately affect many minority ethnic groups and the poorest in society.^13–16^ To inform interventions and service provision, large studies in diverse urban areas are required.^12^

To the best of our knowledge, REACH is the largest and most comprehensive contemporary study of mental health among young people from diverse inner-city areas in the UK. The highly diverse and representative REACH cohorts are drawn from two of the most densely populated and socioeconomically and ethnically diverse boroughs in England,^17–19^ Lambeth and Southwark, London. These boroughs consistently rank among the 20% most deprived boroughs in the country.^17–19^ The prevalence of adult mental disorders is around two times higher in these boroughs compared with national estimates.^12^ REACH provides important new data about the development and trajectories of mental health problems in diverse groups and investigates why, despite similar experiences and circumstances, some young people develop mental health problems whereas others do not. In doing so, REACH will inform the development of interventions to promote mental health and prevent mental health problems in young people from all backgrounds.

## Who is in the cohrt?

### Study design

**Figure 1.**
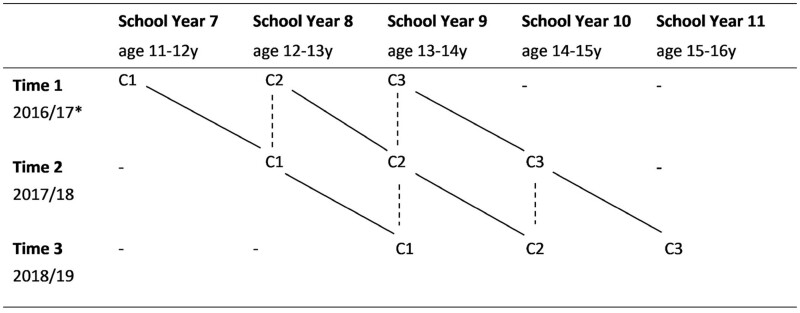
REACH (Resilience, Ethnicity, and AdolesCent Mental Health) study design, accelerated cohort study. C1, Cohort 1; C2, Cohort 2; C3, Cohort 3. Dashed lines indicate points where cohorts overlap. C1, C2, and C3 overlap at School Year 8; C2 and C3 overlap at School Year 9; C2 and C3 overlap at School Year 10. *For one participating school, baseline data collection was deferred by a year so data were collected in 2018 (T1), 2019 (T2), 2020 (T3)

REACH is an accelerated cohort study (Figure 1) comprising three cohorts of young people recruited from 12 state-funded mainstream secondary (high) schools in Lambeth and Southwark, London, UK. The cohorts were recruited and first assessed at age 11-12 years (Cohort 1, school year 7), 12-13 years (Cohort 2, school year 8) and 13-14 years (Cohort 3, school year 9), i.e. in Key Stage 3. Schools were selected to be representative of the 38 mainstream secondary schools within the two boroughs based on (i) the proportion of students eligible for free school meals (a marker of household socioeconomic disadvantage) and (ii) the proportion of students in minority ethnic groups. In the 12 participating schools, all students in school years 7-9 were invited to participate (*n* = 4945).

In line with similar studies,^20–22^ informed consent (for those aged 16 and over) and assent (for those aged under 16) was obtained from all participants using the following procedures. Approximately 2 weeks prior to data collection, researchers visited the school to deliver assemblies on REACH and to distribute information packs for parents and young people. Information was also made available via the study website and, where possible, school websites and mailing lists. Parents were asked to return a form or contact the school or research team if they did not want their child to take part. On the day of assessment, students received further verbal and written information from researchers and, if happy to take part, provided written assent before completing a computerized battery of validated questionnaires, in class, on study tablet computers. All baseline questionnaires were administered between February 2016 and January 2018. Those who completed the baseline questionnaires (i.e. ‘Part 1’) were then invited to take part in a nested sub-study (i.e. ‘Part 2’), comprising a face-to-face interview, hair sample (for cortisol), and cognitive tasks, which we aimed to complete with a subsample of the cohort. Written information about the sub-study was distributed to these young people and to their parents/carers; parents/carers were asked to provide written consent for their child to take part. On the day of Part 2 assessments, young people were given further verbal and written information by trained researchers and provided written assent before taking part. All study procedures were approved by the Psychiatry, Nursing and Midwifery Research Ethics Subcommittee (PNM-RESC), King’s College London (ref: 15/162320).

**Figure 2.**
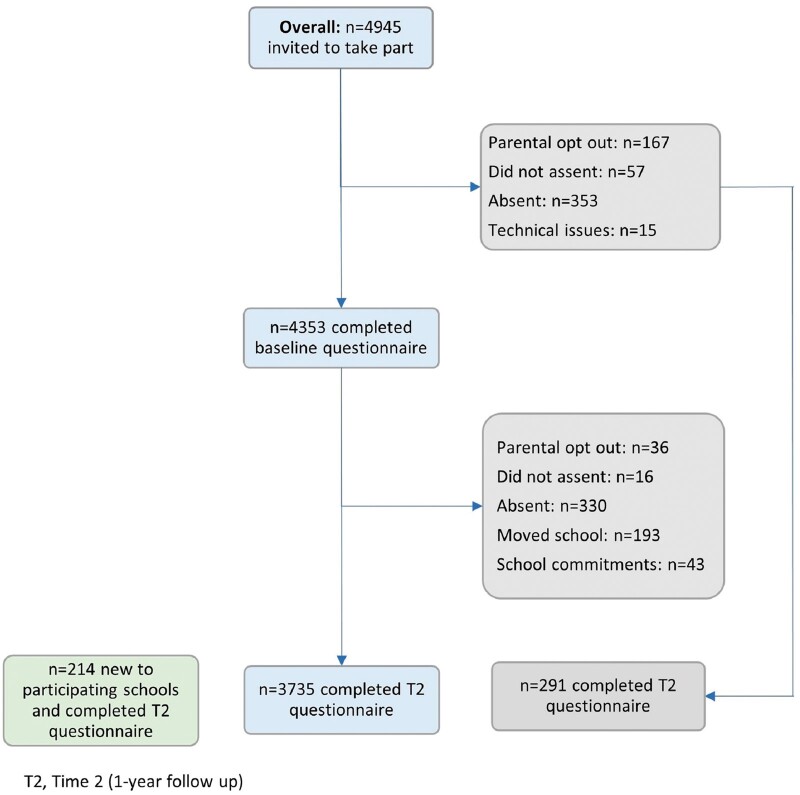
Participation flowchart, cohorts combined. (See Supplementary File 1 for cohort-specific participation flowcharts, available as Supplementary data at *IJE* online.)

Of the 4945 eligible students who were invited to participate in Part 1 at baseline, 4353 (88.0%) completed the baseline questionnaire (Figure 2): Cohort 1: *n* = 1593 (88.0%); Cohort 2: *n* = 1421 (90.0%); Cohort 3: *n* = 1339 (86.1%). Of those who did not take part, 353 (7.2%) were persistently absent, 167 (3.4%) parents refused, 57 (1.2%) young people refused, and 15 (0.3%) provided insufficient data due to technical issues with the study tablet. Of those who participated in Part 1, 85% were from minority ethnic groups and 24% were eligible for free school meals (Table 1). The REACH cohorts are highly representative of the target population (Table 1).^23^

**Table 1:** Comparison of REACH (Resilience, Ethnicity, and AdolesCent Mental Health) cohorts and target population demographics at baseline

	Target population (Key Stage 3 pupils in Lambeth and Southwark)a		REACH cohorts							
	All		All		Cohort 1		Cohort 2		Cohort 3	
	**(*n* = 15** **433)**		(*n* = 4353)		(*n* = 1593)		(*n* = 1421)		(*n* = 1339)	
	*n*	%	*n*	%	*n*	%	*n*	%	*n*	%
Gender										
Boys	7799	50.5	2138	49.1	778	48.8	701	49.3	659	49.2
Girls	7634	49.5	2215	50.9	815	51.2	720	50.7	680	50.8
Receiving free school meals										
No	11 544	74.8	3137	76.3	1142	76.5	1015	75.4	980	76.9
Yes	3889^b^	25.2^b^	976	23.7	351	23.5	331	24.6	294	23.1
Ethnic group										
Black African	4195	27.2	1113	25.6	383	24.0	374	26.3	356	26.6
Black Caribbean	2160	14.0	719	16.5	234	14.7	257	18.1	228	17.0
Other Black	714	4.6	127	2.9	49	3.1	46	3.2	32	2.4
Mixed ethnic groups	1925	12.5	617	14.2	230	5.3	199	4.6	188	4.3
Indian, Pakistani, Bangladeshi	497	3.2	181	4.2	79	5.0	57	4.0	45	3.4
White British	2528	16.4	667	15.3	285	17.9	185	13.0	197	14.7
Non-British White	1671	10.8	626	14.4	224	5.1	200	4.6	202	4.6
Other/unknown	1339	8.7	303	7.0	109	6.8	103	7.3	91	6.8

a Lambeth and Southwark Key Stage 3 (KS3) demographics obtained, by application, from the National Pupil Database Spring 2017 School Census.

b Free school meals (FSM) data for Lambeth and Southwark are not available by Key Stage, or by school year group, so the data presented here (percentage of Lambeth and Southwark pupils receiving free school meals) is for Key Stage 3 (i.e. Years 7–9, age 11–14) and Key Stage 4 (Years 10-11, age 14–16) pupils combined (25.2%, 2017 Spring Census. Source: Department for Education^23^) and we used this to calculate an estimated frequency. Table adapted, with permission, from Knowles *et al*., 2021.^25^

For Part 2, our *a priori* target was to interview, at two time points, 552 young people and for this subsample to be broadly representative of the target population on core demographics (i.e. gender, ethnic group, age/cohort, and free school meals status) and with ∼25% experiencing mental health problems [i.e. a score of 18+ on the Strengths and Difficulties Questionnaire (SDQ) based on responses to the Part 1 questionnaire]. Of those who completed baseline Part 1 questionnaires (*n* = 4353), consent for Part 2 was obtained for 1060 (21.4%) young people. To achieve our target, we stratified these 1060 participants by core demographics and mental health status and selected young people at random from within these strata to complete Part 2. Baseline interviews were completed with 803 young people. This exceeded our target sample size, allowing for attrition at follow-up interviews.

## How often have they been followed up?

### Part 1: in-class questionnaires

In the first phase of REACH (i.e. adolescence), the cohorts are assessed annually at three time points. Baseline (T1) and 1-year (T2) and 2-year (T3) follow-up assessments have been completed. T3 data cleaning is ongoing. As of May 2020, all 12 schools remain in the study. Of the 4353 young people who completed the T1 questionnaire, 3735 (85.8%) completed the T2 questionnaire. T1-to-T2 attrition was 14.2%. Reasons for non-participation at T2 (among those who took part at T1) were: persistent absence despite repeated visits by researchers [*n* = 330 (7.6%)]; present but unable to take part because of competing commitments at school [*n* = 43 (1.0%)]; moved to a non-participating school [*n* = 193 (4.5%)]; parents refused [*n* = 36 (0.8%)]; young person refused (*n* = 16 (0.4%)]. (See Supplementary File 1, available as Supplementary data at *IJE* online, for a breakdown of these numbers by cohort.) Compared with those who took part at T2, non-participants at T2 were more likely to be boys, from poorer households (i.e. eligible for free school meals) and to have had mental health difficulties at T1 (Table 2). However, the magnitude of these differences is small, and the cohorts remain highly representative of the target population (Tables 1 and 2). Due to the study design and the nature of doing research in schools, some of those who were not reassessed at T2 (i.e. the 14% T1-to-T2 attrition) return to the study and are reassessed at T3 (e.g. those who were absent from school at T2 but not at T3). To date, 4005 (92% of those who took part at T1) have completed at least one follow-up (i.e. T2 and/or T3).

**Table 2:** Baseline characteristics of those who did and did not participate at Time 2 (T2, 1-year follow up), overall and by cohort

	All				Cohort 1				Cohort 2				Cohort 3			
	T2 participation status				T2 participation status				T2 participation status				T2 participation status			
Baseline characteristic, *n:* %	Did not participate		Participated		Did not participate		Participated		Did not participate		Participated		Did not participate		Participated	
	(*n* = 618)		(*n* = 3735)		(*n* = 204)		(*n* = 1389)		(*n* = 206)		(*n* = 1215)		(*n* = 208)		(*n* = 1131)	
Girls	274	44.2	1939	51.9	91	44.6	723	52.0	89	43.2	630	51.9	94	45.2	586	51.8
Boys	344	55.7	1796	48.1	113	55.4	666	48.0	117	56.8	585	48.2	114	54.8	545	48.2
Eligible for free school meals																
Yes	164	28.1	813	23.0	56	29.3	295	22.7	53	27.5	278	24.1	55	27.5	240	22.4
No	420	71.9	2716	23.0	135	70.7	1007	77.3	140	72.5	875	75.9	145	72.5	834	77.7
Ethnic group																
Black African	134	21.7	978	26.2	45	22.1	338	24.3	38	18.5	334	27.5	51	24.5	306	27.1
Black Caribbean	132	21.4	582	15.6	41	20.1	188	13.5	42	20.1	213	17.5	48	23.1	181	16.0
Other Black	20	3.2	110	3.0	8	3.9	44	3.2	8	3.9	39	3.2	4	1.9	27	2.4
Mixed White and Black	66	10.7	320	8.6	21	10.3	126	9.0	22	10.7	98	8.1	23	11.1	96	8.5
Other Mixed	35	5.7	208	5.6	11	5.4	80	5.8	10	4.9	73	6.0	14	6.7	55	4.9
Indian, Pakistani, Bangladeshi	19	3.1	161	4.31	8	3.9	70	5.0	6	2.9	51	4.2	5	2.4	40	3.5
Latin American	36	5.8	184	4.9	9	4.4	50	3.6	19	9.2	61	5.0	8	3.9	73	6.5
White British	60	9.7	579	15.5	22	10.8	250	18.0	13	6.3	162	13.3	25	12.0	167	14.8
non-British White	52	8.4	378	10.1	22	10.8	152	10.9	18	8.7	111	9.1	12	5.8	115	10.2
Any other/Unknown	64	10.4	235	6.3	17	8.3	91	6.6	29	14.1	73	6.0	18	8.7	71	6.3
Mental health difficulties^a^																
Yes	133	22.2	668	18.3	51	25.5	254	18.7	42	21.5	217	18.3	40	19.7	197	17.8
No	465	77.8	2981	81.6	149	74.5	1106	81.3	153	78.5	967	81.7	163	80.3	908	82.2
Continuous variables, mean: SD																
SDQ total difficulties scores	12.8	5.8	12.1	5.8	13.6	6.1	12.0	5.9	13.0	5.7	12.1	5.7	12.0	5.6	12.1	5.7
SDQ internalizing scores	5.4	3.5	5.6	3.5	5.8	3.7	5.6	3.5	5.6	3.4	5.7	3.4	5.0	3.4	5.6	3.5
SDQ externalizing scores	7.4	3.8	6.5	3.6	7.8	3.9	6.4	3.7	7.5	3.8	6.5	3.5	7.0	3.8	6.6	3.5

SDQ, Strengths and Difficulties Questionnaire, a widely used and validated self-report measure of behavioural and emotional difficulties for 11–17 year olds (see Supplementary File, available as Supplementary data at *IJE* online for more information on measures).

a SDQ total difficulties score >17. Percentages are column percentages and may not add up to 100 due to rounding.

T4 is ongoing, online, and is generating information about the heterogeneous impacts of the COVID-19 pandemic, including school closures and other social distancing measures, on young people from disadvantaged and diverse backgrounds.^24^

### Part 2: face-to-face interview

In the first phase of REACH, Part 2 is conducted at two time points, approximately ∼1 year apart. Of the 803 young people who completed a baseline Part 2 assessment, 598 (74.5%) have completed the 1-year follow up, exceeding our initial target of 552.

## What has been measured?

Table 3 provides a broad overview of data collected. Briefly, the Part 1 in-class questionnaire comprises validated and widely used measures and collects information on mental health, putative risk and protective factors, demographics, and social circumstances. Supplementary File 3, available as Supplementary data at *IJE* online, provides a more detailed breakdown of the types of information collected and the measures used at each time point and in each part of the study. The questionnaire takes ∼1 h to complete, and trained researchers (around 1 per 6 participants) are present in all sessions to answer questions. The Part 2 sub-study collects more in-depth information (i.e. frequency, severity, duration, impact, and detailed descriptions of experiences, including support sought/received at the time) on mental health, putative risk and protective factors, and potential mechanisms linking risk and protective factors and mental health [e.g. neurocognition, social cognition, hypothalamic-pituitary-adrenal (HPA) axis activation (hair cortisol)]. All interviews are administered by two trained researchers and take on average 2 h to complete. In addition, linkage of REACH data to data routinely collected by schools for the National Pupil Database (i.e. academic attainment and progress, attendance, exclusions, etc.) is ongoing. In future waves, we will explore the feasibility of linkages to medical records and collection of samples for DNA. Moreover, the REACH cohorts and our ongoing partnerships with schools provide a strong platform for innovative nested studies to further examine the mechanisms linking socioenvironmental risk and protective factors with mental health. For example, ∼480 young people from the REACH cohorts and schools are taking part in an innovative longitudinal study using virtual reality to investigate the mechanisms linking difficult experiences (e.g. bullying, threat, violence) and state paranoia (i.e., context specific paranoia).

**Table 3:** Broad overview of data collected in REACH (Resilience, Ethnicity, and AdolesCent Mental Health)

	Part 1, in-class questionnaires			Part 2, interviews	
	T1	T2	T3	T1	T2
Demographics and social circumstances					
Age	✓	✓	✓		
Gender	✓	✓	✓		
Ethnic group	✓	✓	✓		
Eligibility for free school meals	✓	✓	✓		
Languages spoken	✓	✓	✓		
Religion	✓	✓	✓		
Frequency of worship	✓	✓	✓		
Place of birth	✓	✓	✓		
Parents' place of birth	✓	✓	✓		
Parents' employment status	✓	✓	✓		
Family Affluence Scale	✓	✓	✓		
Household/family structure	✓	✓	✓		
Mental health					
Strengths and Difficulties Questionnaire (SDQ)	✓	✓	✓		
Generalized Anxiety Disorder Scale (GAD-7)	✓		✓		
Short Mood and Feelings Questionnaire (SMFQ)	✓		✓		
Self-harm	✓	✓	✓	✓	✓
Behaviour checklist	✓	✓	✓	✓	✓
Adolescent Psychotic Symptom Screener	✓	✓	✓	✓	✓
Development and Adolescent Well-being Asssessment (DAWBA)				✓	✓
Difficult experiences, life events					
Bullying	✓	✓	✓	✓	✓
Adolescent-appropriate Major Life Events Checklist	✓	✓	✓		
Homelessness	✓	✓	✓		
School moves and exclusions	✓	✓	✓		
Home moves	✓	✓	✓		
Migration	✓	✓	✓		
Juvenile Victimisation Questionnaire				✓	✓
Adolescent-appropriate Life Events Checklist	✓	✓	✓	✓	✓
Parents' and siblings’ mental health	✓	✓	✓		
Parents' physical health	✓	✓	✓		
Discrimination	✓	✓	✓		
Perception of local neighbourhood	✓	✓	✓		
Gangs	✓	✓	✓		
Contact with police			✓		
Social support, relationships					
Number of friends	✓	✓	✓		
Peer network at school	✓	✓	✓		
Peer and adult confidantes	✓	✓	✓		
Loneliness	✓	✓	✓		
Perceptions of school environment/climate	✓	✓	✓		
Help-seeking	✓	✓	✓	✓	✓
Cultural identity and integration	✓	✓	✓		
Social media/internet use	✓	✓	✓		
Perceived quality of relationships with parents/carers and siblings	✓	✓	✓		
Multidimensional Scale of Perceived Social Support	✓	✓	✓		
Parental Bonding Instrument, short version	✓	✓	✓		
Future aspirations			✓		
Physical health and lifestyle					
Chronic health conditions and disabilities	✓	✓	✓		
Self-perceived health status	✓	✓	✓		
Physical activity questionnaire for older children (PAQ-C)	✓	✓	✓		
Weekly frequency of breakfast consumption	✓	✓	✓		
Child Report Sleep Patterns Questionnaire	✓	✓	✓		
Smoking, alcohol, and substance use	✓	✓	✓		
Mechanisms					
Children’s Coping Strategies Checklist	✓	✓	✓		
Responses to Stress Questionnaire				✓	✓
Children’s Attributional Style Questionnaire-Revised (CASQ-R)				✓	✓
Shortened Wechsler Abbreviated Scale of Intelligence				✓	✓
Child and Adolescent Mindfulness Measure				✓	✓
Brief Core Schema Scales				✓	✓
Emotion Recognition-40 (ER-40) Test				✓	✓
Cortisol (hair sample)				✓	✓

T1, Time 1 (baseline); T2, Time 2 (1-year follow-up); T3, Time 3 (2-year follow-up);

## What has it found?

Baseline data on the extent and nature of mental health problems among young people in inner-city London are published^25^^,^^26^ and core findings from later waves are under review or in press. Two key findings are summarized below.

### Extent and nature of mental health problems

**Figure 3.**
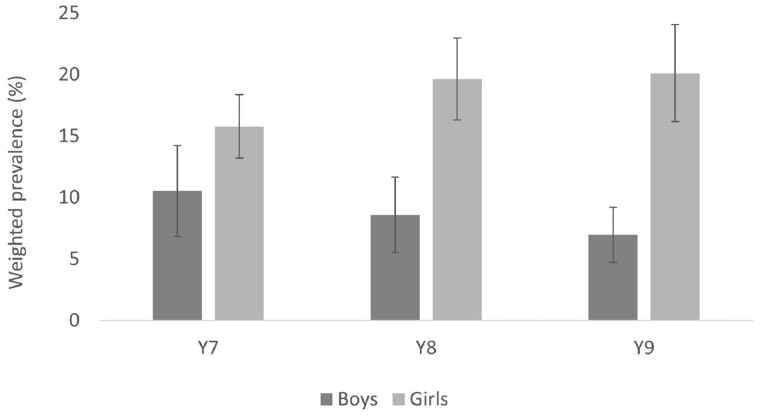
Prevalence of anxiety, by gender and cohort. (Figure reproduced, with permission, from Knowles et al., 2021)

**Figure 4.**
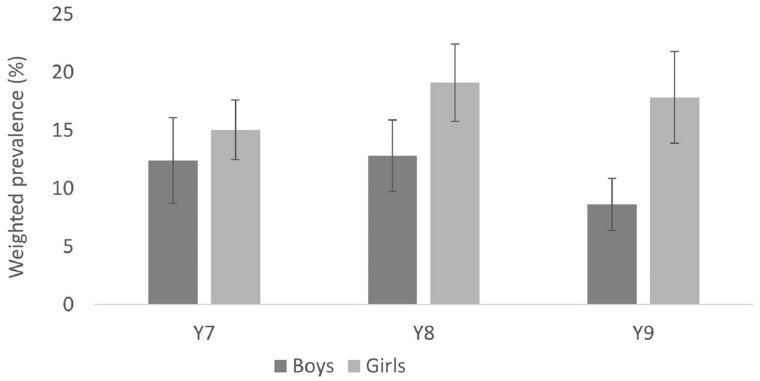
Prevalence of depression, by gender and cohort. (Figure reproduced, with permission, from Knowles et al., 2021)

Our data suggest that ∼19% of 11–14-year-olds in inner-city London have a mental health problem (weighted prevalence 18.6%, 95% CI: 16.4, 20.8%). This is higher than reported in recent national studies in the UK, including those that have used the same self-report measures (e.g. 12% in Understanding Society^27^). Moreover, comparing our estimates with those from similar ethnically diverse inner-city London studies conducted in the early 2000s, our data suggest that within inner-city London the prevalence of mental health problems has increased over the past 15 to 20 years, among both boys (from ∼10-12% to ∼16%) and girls (from ∼12-17% to ∼21%).^28^^,^^29^ Mental health problems were more common among girls than boys, a difference that was more pronounced in older cohorts (Figures 3 and 4) and among those from economically disadvantaged backgrounds.

Arguably, the most striking observation—with regard to the extent and nature of mental health problems—is that the prevalence of conduct problems in inner-city London schools is around three times higher than reported in a recent national sample [16% (95% CI: 15.2, 17.5) vs 5% (95% CI: 4.6, 5.9]) using the same or similar self-report measures.^26^

### Variations—and similarities—in prevalence of mental health problems across diverse groups

Interestingly, our data suggest many similarities—with some variations—in prevalence of emotional problems (i.e. anxiety, depression) and self-harm by ethnic group. For example, prevalence of mental health problems is similar for White British, Black African, and Black Caribbean groups in REACH.^25^ These similarities are striking, as Black African and Black Caribbean groups in the UK experience, on average, greater social, economic, and environmental adversity than their White British peers. Understanding these similarities, despite variations in the distribution of risk factors, is central to our planned analyses. However, our data also hint at differences in the manifestation of distress across diverse groups. For example, our data suggest that prevalence of conduct/behavioural problems is higher among those from Black African and Black Caribbean backgrounds. Importantly though, our data suggest that modifiable social risk factors, including racial discrimination, contribute to variations in prevalence of conduct disorder by ethnic group.^26^

### Planned analyses

Supplementary File 2, available as Supplementary data at *IJE* online, outlines the core hypotheses and planned analyses for the first phase (Years 1-5) of REACH. Broadly, the planned analyses include: (i) prevalence and trajectories of mental health problems, overall and by social and ethnic group (H1.1-1.2); (ii) associations between socioenvironmental risk factors and mental health trajectories, and the modifying effects of protective factors such as social support (H2.1-2.5); and (iii) mediation of associations between socioenvironmental risk factors and mental health trajectories by social cognition, neurocognition, and HPA axis activation (H3.1-3.2).

## What are the main strengths and weaknesses?

REACH is the largest contemporary UK-based study of mental health among young people from diverse backgrounds. The main strengths of REACH are: high baseline response rates; representative sample; 100% school retention; low participant attrition; the diversity of the cohorts and strong representation of groups that are invariably under-represented in large and national surveys; use of novel data collection methods (i.e. hair cortisol, virtual reality, video diaries) to examine mechanisms and pathways; corroboration of questionnaire data with more in-depth data collected via interviews; biological samples and ongoing data linkages; high frequency of data collection, through critical developmental periods; breadth and depth of information collected, including experiences that are not well documented or understood in the youth mental health literature (e.g. racism); and the accelerated cohort design, which enables rapid collection of data across a wider age range than would be possible in the same time frame with traditional prospective designs and, critically, allows age, period, and cohort effects to be disentangled. In addition, the next phase of REACH will provide robust new information about the impacts of the Covid-19 pandemic on young people from disadvantaged backgrounds and about risk and protective factors for mental health trajectories through the transition to adulthood. Finally, REACH’s extensive and ongoing public engagement programme has engaged over 15 000 local young people, parents, and teachers, and all our engagement materials are available for others conducting school-based research.

Consistent with most large prospective studies, the main potential limitations are attrition bias, missing data, and misclassification due to the use of self-report measures. As presented in Table 2, those who did not participate at T2 differ slightly, on average, compared with those who did take part at T2, in terms of some basic characteristics. They also likely differ, on average, in their risk of mental health problems and in their experiences and social circumstances. This is an important limitation. However, due to the relatively high frequency of data collection in REACH and the school setting for fieldwork, we have been able to collect T3 data on many of those who did not complete the T2 questionnaire. Indeed, around 4000 provided data at two of the first three time points. These data are important for monitoring potential biases arising from missing data and will inform the development of multiple imputation models and inverse probability weights to restore representativeness.^30^ Another key limitation is the potential for misclassification (e.g. mental health status) with the use of self-report measures in Part 1. However, the questionnaire comprises widely used and validated measures, and we will corroborate self-report questionnaire data with data collected via interviews with participants and through ongoing data linkages. Finally, the Part 1 questionnaire is detailed and takes ∼50-60 min to complete. Some students did not finish, so missing data due to item non-response is another potential limitation. Some schools allowed extra time for students who required it, but this was not possible at all schools. Nonetheless, the questionnaire content was deliberately structured to reflect our research priorities, such that mental health measures and information on core risk and protective factors were collected at the start of the questionnaire and lower priority questions were at the end of the questionnaire. Coverage of priority measures at baseline is excellent: for instance, the proportion of students with missing data for the baseline strengths and difficulties questionnaire (SDQ) is <0.1%.

## Can I get hold of the data? Where can I find out more?

We welcome and encourage requests from researchers wishing to access REACH data for specific research projects or collaborations. Our data access policy, which aims to make REACH data as accessible as possible while adhering to legal and ethical principles and protecting the privacy of schools and participants, can be found at [www.thereachstudy.com/information-for-researchers.html]. Further information about REACH is also available on the study website. The application should be submitted to Professor Craig Morgan [craig.morgan@kcl.ac.uk].

## Ethics approval

All study procedures were approved by the Psychiatry, Nursing and Midwifery Research Ethics Subcommittee (PNM-RESC), King’s College London (ref: 15/162320).

## Supplementary data

Supplementary data are available at *IJE* online.

## Author contributions

G.K.: drafted and revised the manuscript; approved the final manuscript; collected, analysed and interpreted the data; contributed to the design of the study; coordinated the study. C.G-A.: revised the manuscript; approved the final manuscript; collected, analysed and interpreted the data; contributed to the design of the study. S.B., R.B., S.D., K.L., D.S., A.O., A.T., L.D.: collected and cleaned the data; critically revised the manuscript; approved the final manuscript. S.W.G.: made substantial contributions to the acquisition and interpretation of data and critically reviewed and approved the final manuscript. V.P.: contributed to the conceptualization and design of the work; interpreted the data; revised the manuscript; approved the final manuscript. U.R.: contributed to the conceptualization and design of the work; critically revised the manuscript; approved the final manuscript. S.H.: contributed to the conceptualization and design of the work; critically revised the manuscript; approved the final manuscript. C.M. was principal investigator and guarantor; conceptualized, designed and secured funding for the study; collected, analysed and interpreted the data; critically revised the manuscript; approved the final manuscript.

## Funding

This work was supported by the UK Economic and Social Research Council (ESRC) Centre for Society and Mental Health (ES/S012567/1); and the European Research Council (ERC) (REACH 648837).

## Supplementary Material

dyac051_Supplementary_Data
