# Correction to: Cohort Profile: Resilience, Ethnicity and AdolesCent mental Health (REACH)

**DOI:** 10.1093/ije/dyac101

**Published:** 2022-05-05

In the originally published version of this manuscript, the group author “Young Persons Advisory Group” was listed sixteenth instead of eleventh.

This error has been corrected. The publisher apologizes for the error.

