# Supplementary material for: Cohort Profile: Resilience, Ethnicity and AdolesCent mental Health (REACH)
Source: Int J Epidemiol. 2022 Mar 28;51(5):e303–13. doi: 10.1093/ije/dyac051 (PMC9557858; doi:10.1093/ije/dyac051)
Supplement: dyac051_Supplementary_Data [file dyac051_supplementary_data.zip › ije-2021-09-1380-File007.docx]

**Cohort 1:** n=1808 invited to take part

Parental opt out: n=89

Did not assent: n=16

Absent: n=107

Technical issues: n=3

n=1593 completed baseline questionnaire

Parental opt out: n=18

Did not assent: n=5

Absent: n=96

Moved school: n=67

School commitments: n=18

n=1389 completed T2 questionnaire

**Cohort 2:** n=1581 invited to take part

Parental opt out: n=36

Did not assent: n=15

Absent: n=104

Technical issues: n=5

n=1421 completed baseline questionnaire

Parental opt out: n=9

Did not assent: n=2

Absent: n=111

Moved school: n=59

School commitments: n=25

n=1215 completed T2 questionnaire

**Cohort 3:** n=1556 invited to take part

Parental opt out: n=42

Did not assent: n=26

Absent: n=142

Technical issues: n=7

n=1339 completed baseline questionnaire

Parental opt out: n=9

Did not assent: n=9

Absent: n=123

Moved school: n=67

School commitments: n=0

n=1131 completed T2 questionnaire
