# Supplementary material for: Cohort Profile: Resilience, Ethnicity and AdolesCent mental Health (REACH)
Source: Int J Epidemiol. 2022 Mar 28;51(5):e303–13. doi: 10.1093/ije/dyac051 (PMC9557858; doi:10.1093/ije/dyac051)
Supplement: dyac051_Supplementary_Data [file dyac051_supplementary_data.zip › ije-2021-09-1380-File008.docx]

**Supplementary file 2 Core Hypotheses and Planned Analyses**

1. **Prevalence and Developmental Trajectories**

**H1.1** At each time point and in each cohort, there will be variations in mental health problems by ethnic group (e.g., more externalising problems and unusual experiences among minority groups)

**H1.2** There will be variations in trajectories of mental health problems over time by ethnic group (e.g., more with a ‘persistent’ trajectory in minority groups)

1. **Risk and Protective Factors**

**H2.1** Socio-environmental exposures at T1 and T2 involving elements of violence and threat (e.g., physical bullying, victim of assault, etc.) will be most strongly associated with a persistent trajectory, overall and in each of the main ethnic groups

**H2.2** The effect of each socio-environmental exposure will be modified by each protective factor (i.e., social network, social support, coping strategies, cognitive ability), such that odds of a persistent trajectory will be highest in those exposed and for whom protective factors are absent or low

**H2.3** Any observed differences between black Caribbean, black African, and white British groups in odds of persistent experiences will be accounted for, at an individual level, primarily by socio-environmental exposures involving violence and threat and discrimination

**H2.4** Each protective factor will have similarly modifying effects in all ethnic groups, but will be less common or lower in black Caribbean and black African groups compared with white British

**H2.5** The odds of a persistent trajectory will be highest in black Caribbean and black African groups when school level own group ethnic density is low

1. **Mechanisms**

**H3.1** Associations between socio-environmental risk factors and trajectories of mental health problems will be at least partially mediated via negative affect (i.e., emotional distress), cognitive schema and biases, and attributional style and, uniquely, these mediation effects will be similar across all ethnic groups

**H3.2** HPA axis activation will be greater in black Caribbean and black African adolescents compared with white British in the context of socio-environmental factors
