# Supplementary material for: Cohort Profile: Resilience, Ethnicity and AdolesCent mental Health (REACH)
Source: Int J Epidemiol. 2022 Mar 28;51(5):e303–13. doi: 10.1093/ije/dyac051 (PMC9557858; doi:10.1093/ije/dyac051)
Supplement: dyac051_Supplementary_Data [file dyac051_supplementary_data.zip › ije-2021-09-1380-File009.docx]

**Supplementary file 3. Study measures and assessments**

**Table S3.1. Part 1 assessments (questionnaire at T1, T2 and T3)**

| **Domain** | | **Questionnaire, item, measure** |
| --- | --- | --- |
| **Basic Information** | | Date of birth  Gender  Postcode  Place of birth of pupil and parents  Language  Self-reported ethnicity  Religion |
| **Mental Health** | | Strengths and Difficulties Questionnaire (Goodman, 1998)  (25 item questionnaire covering presence and impact of emotional problems, conduct problems, hyperactivity and inattention, peer relationship problems, and prosocial behaviour over past 6 months)  Adolescent Psychotic Symptom Screener (Kelleher, 2010)  (8 items on the presence of unusual thoughts and feelings – 2 items on hallucinatory experiences, 6 on delusional experiences – with additional questions on timing, frequency, impact, context, conviction, and a description of the experience)  Short mood and feelings questionnaire (Angold, 1995)*  (13 items on symptoms/experiences of depression in the past 2 weeks)  Generalised Anxiety Disorder Scale (Spitzer, 2006)*  (7 items on symptoms/experiences of anxiety in the past 2 weeks)  1 item on self-harm from the Development and Adolescent Wellbeing Assessment (DAWBA) (Goodman, 2000) †  6 items on troublesome behaviour from the DAWBA (Goodman, 2000) |
| **Risk** | |  |
|  | Socioeconomic Status | Family Affluence Scale (Wardle, 2002)  (4 item index of common indicators of wealth - ownership of car, computer, number of bedrooms in household, and number of yearly holidays)  Self-reported free school meal status  Parental employment status |
|  | Family Structure | 1 item on who the pupil currently lives with  1 item on reason not living with mum or dad, if applicable |
|  | Family health | 1 item on participant’s physical health  2 items on parents’ physical and mental health  1 item on siblings’ mental health  2 items on participant’s height and weight † |
|  | Life events | Adolescent-appropriate Life Events Checklist (Heubeck & O’Sullivan, 1998)  (16 item checklist; items include death of someone close, parental separation/divorce, serious accident or illness, being a victim of crime)  9 items assessing other difficult experiences, including accidents (family); school exclusions; foster care; family money problems; parental alcohol misuse; migration; homelessness. |
|  | Peer Bullying | Revised Olweus Bully/Victim Questionnaire (Olweus, 1996)  (4 items covering physical, verbal, relational, and cyber bullying) |
|  | Discrimination | 2 items on unfair treatment due to race and religion |
|  | Substance Use | 3 items on smoking  1 item on alcohol use  4 items on cannabis use  1 item on other substance use |
|  | Neighbourhood | 1 item on length of time lived in neighbourhood  4 items on perception of neighbourhood (Smith, 2012) |
|  | Street Gangs | 3 items from the British Crime Survey for 10-15 year olds (Milard, 2010), and 3 items from the Eurogang Survey (Medina, 2013) † |
| **Protective** | |  |
|  | Social Networks | 1 item on number of friends  2 items on peer and adult confidants  1 item on loneliness  1 item on best friends in own year group at school (for Social Network Analysis)  2 items on internet use |
|  | Family Relationships and Social Support | Parental Bonding Instrument, short version (Parker , 1979)  (12-item questionnaire on parental care and parental control)  3 items on perceived quality of relationships with parents/carers and siblings  Multidimensional Scale of Perceived Social Support (Zimmet, 1990)  (12 item questionnaire on perceived support from family, friends, and others) |
|  | Help & Support | 11 + 3 items on contact with a range of formal helping agents for emotional or behavioural difficulties (e.g. school counsellors, mental health professionals) (Green, 2005) † |
|  | School Environment | 5 items on perception of school environment/climate (McNeelyet al., 2002) † |
|  | Cultural Integration | 2 items on how many friends are from the pupil’s ethnic group and how many from other ethnic groups (Bhui et al., 2005a and 2005b; Berry, 2004) |
|  | Coping Strategies | Children’s Coping Strategies Checklist (Ayers et al., 1996)  (26 item questionnaire assessing four types of coping: distraction, support seeking, active, avoidant) |
| **Mechanisms** | |  |
|  | Sleep | Child Report Sleep Patterns Questionnaire (Meltzer, 2013) ^‡^  (9 item questionnaire on frequency, duration, and quality of sleep) |
|  | Physical activity | Physical Activity Questionnaire for Children (Kowalski, 2004) ^‡^  (9 items on frequency, intensity, and types of activities in past seven days) |
| *T1 and T3 only; †at T1, not administered at two (originally pilot) schools; ^‡^at T1, modified/short version of measure used at two (originally pilot) schools (full version used at 10 schools). | | |

**Table S3.2. Part 2 assessments (interview and reasonsing tasks at two time points)**

| **Domain** | | **Questionnaire, Interview, Task** |
| --- | --- | --- |
| **Mental Health** | | Adolescent Psychotic Symptom Screener (Kelleher, 2010)  (8 items on the presence of unusual thoughts and feelings – 2 items on hallucinatory experiences, 6 on delusional experiences – with additional questions on timing, frequency, impact, context, a description of the experience)  Child and Adolescent Self-Harm Questionnaire (Madge, 2008) ^‡^  (7 items on presence and nature of self-harm behaviours)  Development and Adolescent Wellbeing Assessment (Goodman, 2000) †  (structured interview on depression, anxiety, conduct disorder, PTSD) |
| **Risk** | |  |
|  | Victimisation and other adversities | Revised Olweus Bully/Victim Questionnaire (Olweus, 1996)  (4 items covering physical, verbal, relational, and cyber bullying, with additional questions on timing, frequency, severity, impact, support, and description of experience)  Juvenile Victimisation Questionnaire (Finkelhor, 2011)  (Semi-structured interview on four domains of victimisation: crime, maltreatment, peer, and witnessing violence)  Adolescent-appropriate Life Events Checklist (Heubeck & O’Sullivan, 1998)  (16 item checklist; items include death of someone close, parental separation/divorce, serious accident or illness, being a victim of crime) |
| **Mechanisms** | |  |
|  | Social cognition | Emotion Recognition-40 Test (ER-40; Gur, 2001) †  (computerised task assessing facial emotion recognition)  Children’s Attributional Style Questionnaire-Revised (CASQ-R) (Kaslow, 1991) †  (24 item structured questionnaire on attributional styles: internal/external; stable/unstable; global/specific)  Brief Core Schema Scales (Fowler, 2006)  (24 item measure of core schematic beliefs about self and others) |
|  | Cortisol | Hair sample  (two small hair samples, approx. 3 cm in length, from the back of the scalp to assess chronic stress response [HPA axis activation]) |
|  | Mindfulness | Child and Adolescent Mindfulness Measure (Greco, 2011)  (10 item questionnaire on dimensions of mindfulness [i.e., present moment awareness]) |
|  | Resilience | Responses to Stress Questionnaire (RSQ) (Connor-Smith, 2000) †  (structured questionnaire assessing coping and involuntary responses to stress) |
| **Other** | |  |
|  | IQ | Shortened Wechsler Abbreviated Scale of Intelligence – Second Edition (WASI-II) (two tasks assessing vocabulary and matrix reasoning and provides a reliable measure of general cognitive ability) |
| *T1 and T3 only; †at T1, not administered at two (originally pilot) schools; ^‡^at T1, modified/short version of measure used at two (originally pilot) schools (full version used at 10 schools). | | |

**Supplementary references (measures)**

Angold A, Costello EJ, Messer SC, Pickles A. Development of a short questionnaire for use in epidemiological studies of depression in children and adolescents. *International Journal of Methods in Psychiatric Research* 1995; **5**(4): 237-49.

Berry JW. Psychology of group relations: cultural and social dimensions. Aviat Space Environ Med 2004; 75(7 Suppl.):C52–57.

Bhui K, Lawrence A, Klineberg E et al. Acculturation and health status among African-Caribbean, Bangladeshi and White British adolescents: –validation and findings from the RELACHS study. Soc Psychiatry Psychiatr Epidemiol 2005(a);40:259–66.

Bhui K, Stansfeld S, Head J et al. Cultural identity, acculturation, and mental health among adolescents in east London’s multiethnic community. J Epidemiol Community Health 2005(b);59:296–302.

Connor-Smith, J. K., Compas, B. E., Wadsworth, M. E., Thomsen, A. H., & Saltzman, H. (2000). Responses to stress in adolescence: measurement of coping and involuntary stress responses. Journal of Consulting and Clinical Psychology, 68(6), 976.

Cullen AE, Fisher HL, Roberts RE, Pariante CM, Laurens KR. Daily stressors and negative life events in children at elevated risk of developing schizophrenia. Br J Psychiatry. 2014 May;204:354-60. PubMed PMID: 24627296. Epub 2014/03/15. eng.

Finkelhor D, Hamby S, Turner H, Ormrod R. The Juvenile Victimisation Questionnaire: 2nd Revision. Durham, NH: Crimes Against Children Research Centre; 2011.

Fowler D, Freeman D, Smith B, Kuipers E, Bebbington P, Bashforth H, et al. The Brief Core Schema Scales (BCSS): psychometric properties and associations with paranoia and grandiosity in non-clinical and psychosis samples. Psychol Med. 2006 Jun;36(6):749-59

Goodman R, Ford T, Richards H, Gatward R, Meltzer H. The Development and Well-Being Assessment: description and initial validation of an integrated assessment of child and adolescent psychopathology. *J Child Psychol Psychiatry* 2000; **41**(5): 645-55.

Goodman R, Meltzer H, Bailey V. The Strengths and Difficulties Questionnaire: a pilot study on the validity of the self-report version. Eur Child Adolesc Psychiatry 1998; 7(3): 125-30.

Greco LA, Baer RA, Smith GT. Assessing mindfulness in children and adolescents: development and validation of the Child and Adolescent Mindfulness Measure (CAMM). Psychological assessment. 2011 Sep;23(3):606-14.

Green H, McGinnity Á, Meltzer H, Ford T, Goodman R (2005). Mental health of children and young people in Great Britain, 2004, Summary Report. Palgrave MacMillan: London

Gur RC, Ragland JD, Moberg PJ, Turner TH, Bilker WB, Kohler C, Siegel SJ, Gur RE (2001). Computerized neurocognitive scanning: I. Methodology and validation in healthy people. Neuropsychopharmacology, 25: 766–776

Heubeck B, O’Sullivan C. An exploration into the nature, frequency and impact of school hassles in the middle school years. Aust Psychol 1998; 33: 130–7

Kaslow NJ, Nolen-Hoeksema S (1991). Children's Attributional Questionnaire—Revised. Unpublished manuscript, Emory University, Atlanta, GA.

Kelleher I, Cannon M. Psychotic-like experiences in the general population: characterizing a high-risk group for psychosis. Psychol Med. 2011 Jan;41(1):1-6.

Kowalski KC, Crocker PRE, Donen RM. The Physical Activity Questionnaire for Older Children. Saskatoon, Canada: University of Saskatchewan; 2004.

Madge N, Hewitt A, Hawton K, de Wilde EJ, Corcoran P, Fekete S, et al. Deliberate self-harm within an international community sample of young people: comparative findings from the Child & Adolescent Self-harm in Europe (CASE) Study. J Child Psychol Psychiatry. 2008 Jun;49(6):667-77.

McNeely CA, Nonnemaker JM, Blum RW (2002). Promoting school connectedness: Evidence from the national longitudinal study of adolescent health. Journal of School Health, 72(4), 138-146

Medina J, Aldridge J, Shute J, Ross A (2013). Measuring gang membership in England & Wales: A latent class analysis with Eurogang survey questions. Criminology & Penology, 10(5), 591-605

Meltzer LJ, Avis KT, Biggs S, Reynolds AC, Crab-tree VM, Bevans KB (2013). The Children's Report of Sleep Patterns (CRSP): a self-report measure of sleep for school-aged children. Journal of Clinical Sleep Medicine, 9(3), 235-245

Millard B, Flatley J (ed.) (2010). Experimental statistics on victimisation of children aged 10 to 15: findings from the British Crime Survey for the year ending December 2009 England and Wales, Home Office Statistical Bulletin 11/01, London: Home Office. <http://www.homeoffice.gov.uk/rds/pdfs10/hosb1110.pdf>

Olweus D. The Revised Olweus Bully/Victim Questionnaire. Bergen, Norway: Research Centre for Health Promotion, University of Bergen; 1996.

Parker G, Tupling H, Brown LB (1979). A parental bonding instrument. British Journal of Medical Psychology, 52(1), 1-10

Smith NR, Clark C, Fahy AE, Tharmaratnam V, Lewis DJ, Thompson C, Renton A, Moore DG, Bhui KS, Taylor SJC, Eldridge S, Petticrew M, Greenhalgh T, Stansfield SA, Cummins S (2012). The Olympic Regeneration in East London (ORiEL) study: protocol for a prospective controlled quasi-experiment to evaluate the impact of urban regeneration on young people and their families. BMJ Open, 2(4), e001840

Spitzer RL, Kroenke K, Williams JB, Lowe B. A brief measure for assessing generalized anxiety disorder: the GAD-7. Archives of internal medicine. 2006 May 22;166(10):1092-7.

Wardle J, Robb K, Johnson F. Assessing socioeconomic status in adolescents: the validity of a home affluence scale. Journal of epidemiology and community health. 2002 Aug;56(8):595-9. PubMed PMID: 12118050. Pubmed Central PMCID: 1732226

Zimet GD, Powell SS, Farley GK, Werkman S, Berkoff KA. Psychometric characteristics of the Multidimensional Scale of Perceived Social Support. J Pers Assess. 1990 Winter;55(3-4):610-7. PubMed PMID: 2280326. Epub 1990/01/01. eng
